# Symbolic iteration method based on computer algebra analysis for Kepler’s equation

**DOI:** 10.1038/s41598-022-07050-5

**Published:** 2022-02-22

**Authors:** Ruichen Zhang, Shaofeng Bian, Houpu Li

**Affiliations:** Department of Navigation, Naval University of Engineering, 717 Jiefang Avenue, Wuhan, China

**Keywords:** Planetary science, Mathematics and computing

## Abstract

The Kepler’s equation of elliptic orbits is one of the most significant fundamental physical equations in Satellite Geodesy. This paper demonstrates symbolic iteration method based on computer algebra analysis (SICAA) to solve the Kepler’s equation. The paper presents general symbolic formulas to compute the eccentric anomaly (*E*) without complex numerical iterative computation at run-time. This approach couples the Taylor series expansion with higher-order trigonometric function reductions during the symbolic iterative progress. Meanwhile, the relationship between our method and the traditional infinite series expansion solution is analyzed in this paper, obtaining a new truncation method of the series expansion solution for the Kepler’s equation. We performed substantial tests on a modest laptop computer. Solutions for 1,002,001 pairs of (*e*, *M*) has been conducted. Compared with numerical iterative methods, 99.93% of all absolute errors *δ*_*E*_ of eccentric anomaly (*E*) obtained by our method is lower than machine precision $\epsilon$ over the entire interval. The results show that the accuracy is almost one order of magnitude higher than that of those methods (double precision). Besides, the simple codes make our method well-suited for a wide range of algebraic programming languages and computer hardware (GPU and so on).

## Introduction

**Figure 1:**
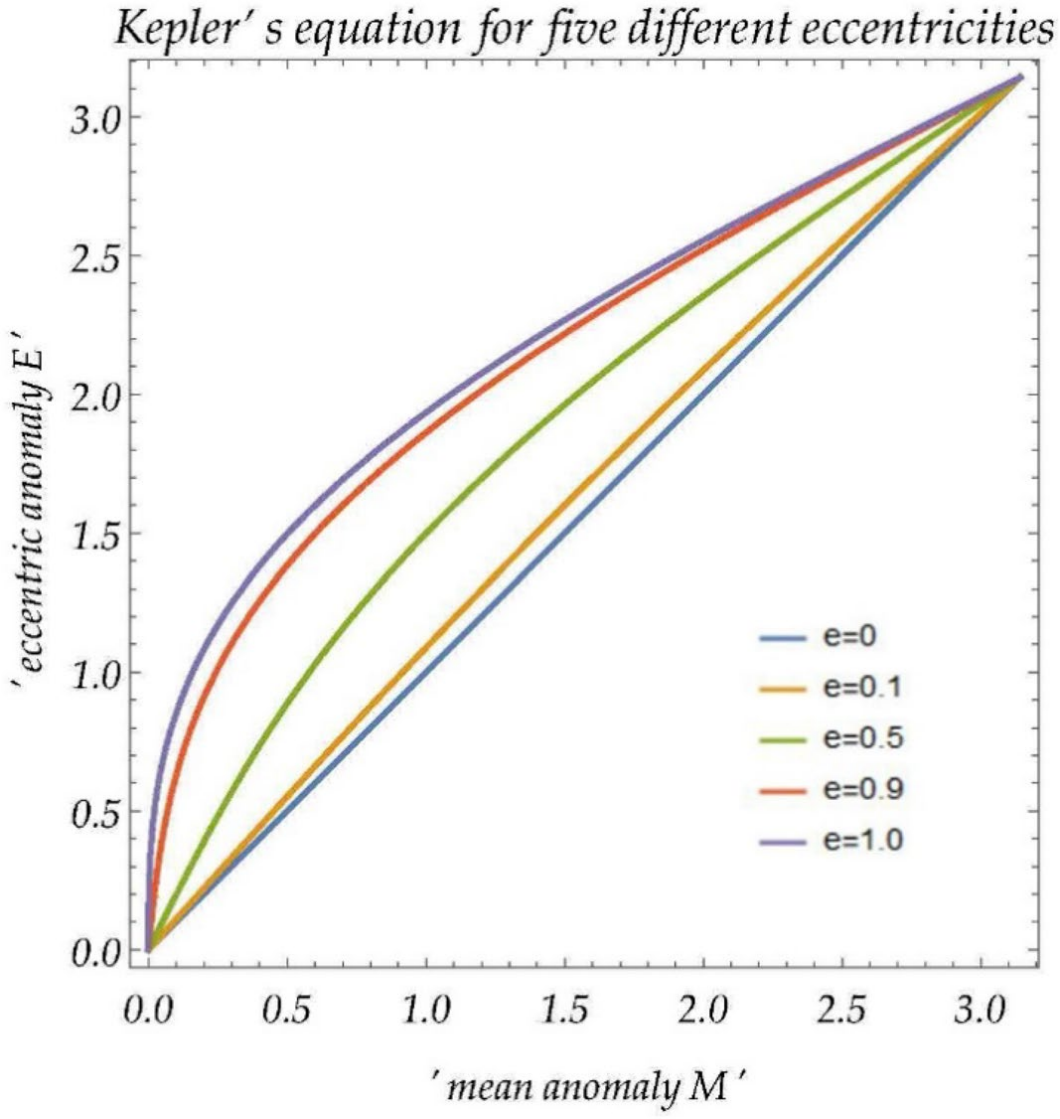
Contours of the Kepler’s equation with different eccentricities ($e=0, 0.1, 0.5, 0.9, 1$).

As one of the fundamental physical equations in Geoscience, the Kepler's equation in elliptical orbits describes a relationship of the eccentric anomaly (*E*), the eccentricity (*e*), and the mean anomaly (*M*)^1^, in which *M* is related to the time. For elliptic orbits, it is shown as Eq. (1), and the contours of the Kepler’s equation with different eccentricities ($e = 0, 0.1, 0.5, 0.9, 1$), are shown in Fig. 1. This paper focuses on the situation of nearly-circular motions ($e \ll 1$). We simplify the problem by limiting $E$ and $M$ within the range of $\left[ {0,{\uppi }} \right]$^2^, as shown in Eq. (2). General Solutions for $\left( {E,M} \right) \in {\mathbb{R}} \times {\mathbb{R}}$ can be easily calculated through ${ }\left( { \pm E + 2n\pi ,{ } \pm M + 2n\pi } \right)$, $n \in {\mathbb{Z}}$.

1 $$E = M + e{ }\sin E$$

2 $$E,M \in \left[ {0,\pi } \right],\quad e \ll 1$$

Brouwer and Clemence^3^, and Danby^4^ have described the principles and applications of this equation for artificial Earth satellites and other celestial bodies in great detail. The enormous researches to solve the Kepler’s equation underline its significance in many fields of Satellite Geodesy (e.g. precise orbit determination, perturbation theory and Earth model calculation), Geophysics and Trajectory Optimization^5–11^. As all the satellite orbits are nearly-circular, this paper focuses on the situation of nearly-circular motions, providing a symbolic iteration method based on computer algebra analysis for Kepler’s equation.

Many scientists have made considerable improvements with high accuracy and low time consumption of various algorithms to solve the equation. Nijenhuis and Albert^1^ combined the Mikkola’s starter algorithm with a higher-order Newton method, requiring only two trigonometric evaluations. Markley and Landis^12^ presented a non-iterative method with four transcendental function evaluations. Colwell^13^ systematically analyzed the traditional solutions for the Kepler’s equation, such as traditional power serious expansion based on Lagrange inversion theorem, and series solutions in Bessel functions of the first kind, which are still the most common methods for Kepler’s equation. Taff and Brennan^14^ explored an extensive starting values and solution techniques for iterative methods, giving the best and simplest starting value algorithm. Odell^15^ provided an iterative process that always gives 4th order convergence, with error less than 7 × 10^−15^ rad. Mikkola^16^ derived a high order iteration formula by adding an auxiliary variable, which could be used both for elliptical and hyperbolic orbits. Sengupta^17^ presented the Lambert W method for the truncation of infinite Fourier–Bessel representations. Calvo and Elipe^18^ compared the quality of starting algorithm for the iterative solution of elliptic Kepler’s equation, giving new optimal starters. Oltrogge and Daniel^19^ derived a solution via families of hybrid and table lookup techniques, achieving the accuracies to machine precision at 3 × 10^−11^. Zechmeister^20^ presented an algorithm to compute the eccentric anomaly without usage of transcendental functions. Tommasini and Olivieri^21^ provided a solution for the inverse of a function based on switching variables followed by spline interpolation.

However, limited by historical scientific conditions, these traditional methods usually contain numerical iterative calculations, such as the standard Newton–Raphson method^2,4^ and the Halley's method^2^, which have to reset the initial values and iterate from the beginning every time the eccentricity *e* changes^22^. Meanwhile, some traditional numerical methods ^4^ contain tremendously complex functions, which costs too much time to compute. In these methods, the CPU time cost by transcendental functions accounts for a large part of the total CPU time consumption^23^. For instance, an iterative method to solve the Kepler's equation usually requires two trig evaluations (sine and cosine) at every iterative step^1^; the CPU time cost increases exponentially with the number of iterations grows. As for the Intel i7 × 920 processor, the calling time of trigonometric functions occupies more than 80% of the total time consumption in measured unmanaged arithmetic^19^. Besides, some traditional formulas are given in the form of concrete numerical values, which lacks generality. Meanwhile, there were also some analytic solutions with a certain tolerance and maximum eccentricity. For example, Markley and Landis^12^ provided a non-iterative method with errors better than 10^−4^ requiring four transcendental functions, containing square-root, cube-root and trigonometric operations. Nijenhuis^1^ further presented a non-iterative method that represented a gain obtained through a starter algorithm, which required two trigonometric evaluations. Although these methods are classic and efficient at that time, their performances are limited by the historical scientific conditions with a relatively poor accuracy.

The current research is mainly aimed at improving the iterative methods and expanding the scope of the application of algorithms. Meanwhile, since the solution for the Kepler’s equation is a traditional problem, the traditional iterative methods are relatively mature. Thus, entering into the new century, there are fewer studies specifically on this problem, letting alone specifically on the circumstances of nearly-circular motions. However, most of the two-body motions in the universe are nearly-circular motions, especially for all satellite motions. The accuracy of the eccentric anomaly (*E*) is the basis and the determinant for the accuracy of satellite orbit determination. Thus, in order to improve the accuracy of the eccentric anomaly (*E*) in the field of satellite geodesy, this paper proposes a solution for Kepler’s equation specifically for the nearly-circular orbits, which improves the accuracy to the order of 10^−17^. In this paper, based on the computer algebra system (Mathematica 12.1 and Python 3.8), we present an analytic method with a general symbolic formula to compute the eccentric anomaly (*E*) without complex numerical iterative computation at run-time for nearly-circular motions. This non-iterative method only requires two trigonometric functions during the whole computation process. Compared with the traditional analytic methods, our analytic method is easy to understand with a higher accuracy and simpler codes; compared with the commonly used traditional iterative methods, our method has higher accuracy and stability; compared with modern solutions for Kepler’s equation, on the basis of retaining the accuracy of the algorithm, our method takes into account the advantages of analytical methods and the simplicity of codes.

## ©

### Basic scheme

The SICAA method is to symbolically iterate the Kepler’s equation in the form of Taylor series expansion without concrete numerical values. Due to the limitation of the computer platform precision, we could control the error $\varepsilon_{E} = E_{n + 1} - E_{n}$ when the residual $\varepsilon_{M} = M_{n + 1} - M_{n}$ is within the accuracy. Nonetheless, the cancellation problem is hard to avoid for the Kepler's equation when the limits of platform precision are pushed. As for this problem, we use the Taylor series expansion for every symbolic operational step, as shown in Eq. (5). The starter of $E$ is shown in Eq. (3). If the variable $e$ in Eq. (4) is regarded as an argument of the function $E\left( e \right)$, the variable $M$ is regarded as the parameter. We suppose ${ }e \ll 1$, so $E_{n + 1} \left( e \right)$ is expanded at $e{ } = { }0$ by Taylor Series expansion, and then Eq. (4) can be expanded as Eq. (5).

3 $$E_{0} \approx M\quad \left( { e \ll 1} \right)$$

4 $$E_{n + 1} \left( {e,M} \right) \approx M + e\sin E_{n} \left( {e,M} \right)$$

5 $$E_{n + 1} \left( {e,M} \right) \approx E_{n + 1} \left( {0,M} \right) + E_{n + 1}^{^{\prime}} \left( {0,M} \right)e + \frac{{E_{n + 1}^{^{\prime\prime}} \left( {0,M} \right)}}{2!}e^{2} + \ldots + \frac{{E_{n + 1}^{k} \left( {0,M} \right)}}{k!}e^{k}$$

where $$n = 0,1,2, \ldots \;k = 0,1,2, \ldots 18$$.

The final analytic formula to calculate the eccentric anomaly obtained by our SICAA method is shown in Eq. (6), where series is extended to infinitely small quantity $O\left[ e \right]^{18}$. The specific coefficients of $\sin nM$ are shown in supplementary material S.4.

6 $$E = f\left( {e,M} \right) = M + a_{1} \sin M + a_{2} \sin 2M + a_{3} \sin 3M + a_{4} \sin 4M + a_{5} \sin 5M + \cdots + a_{i} \sin nM$$

It can be found that our analytic solution is really intuitive, and the process of the SICAA method is easy to conduct and simple to understand.

### Convergence criteria and convergence domain

According to the convergence rule, we need to discuss the convergence of Eq. (4). Thus, we rearrange Eq. (4). The function $f_{1} \left( E \right)$ is defined as $f_{1} \left( E \right) = E$. The function $f_{2} \left( E \right)$ is defined as $f_{2} \left( E \right) = M + e\sin E$. When $e < 1$, for $\exists E_{a} \in \left[ {0,\pi } \right], \exists E_{b} \in \left[ {0,\pi } \right]$, so $\left| {\cos E_{b} } \right| \le 1$ and eventually we can get the Eq. (7).

7 $$\frac{{\left| {f_{2}^{^{\prime}} \left( {E_{b} } \right)} \right|}}{{\left| {f_{1}^{^{\prime}} \left( {E_{a} } \right)} \right|}} = \frac{{\left| {e\cos E_{b} } \right|}}{\left| 1 \right|} = \left| {e\cos E_{b} } \right| < 1$$

The above iterative equation converges at $E \in \left[ {0,\pi } \right]$ and $e < 1$. The convergence rate (also called error disappearance rate) is equivalent to $\alpha^{n}$. The specific formula of $\alpha$ is shown in Eq. (8).

8 $$\alpha \approx \frac{{E_{2} - E_{1} }}{{E_{1} - E_{0} }}$$

### The relationship between the SICAA method and the Bessel function

The traditional series expansion of the eccentric anomaly^9,13^ is shown as the Eqs. (24) and (25) in “Mathematics essence of our method and relationship with traditional Fourier–bessel series” section. By comparing the coefficients of our method (SICAA) and those of Fourier–Bessel series expansion of the eccentric anomaly, the relationship between SICAA and the Bessel function are shown in Eqs. (9) and (10).

9 $$E_{{{\text{SICAA}}}} \approx M + 2\mathop \sum \limits_{k = 1}^{{k_{max} }} \frac{1}{k}J_{k}^{{\left\lceil {k_{max} - k + 1} \right\rceil }} \left( {ke} \right)\sin kM$$

10 $$J_{k}^{{\left\lceil {k_{max} - k + 1} \right\rceil }} \left( {ke} \right) = \mathop \sum \limits_{j = 0}^{{\left\lceil {k_{max} - k + 1} \right\rceil }} \left( { - 1} \right)^{j} \frac{{\left( \frac{ke}{2} \right)^{k + 2j} }}{{j!\left( {k + j} \right)!}}$$

where $\left\lceil * \right\rceil$ is the Ceiling function. From the Eqs. (9) and (10), we can see that the coefficients of the SICAA method are the truncation ($\left\lceil {k_{max} - k + 1} \right\rceil$) of the first kind infinite Bessel function.

## Variants and refinements

As mentioned above, the code of SICAA method is simple and easy to implement with strong expansibility. We also present some generalized types of our SICAA method.

### The Newton-like SICAA method

The convergence of the Eq. (4) is linear. Based on Newton numerical iterative theory, the convergence of the Eq. (11) is quadratic. Our algorithm can serve derivatives and starters at any stage in a symbolic way. The quadratic convergence could get the accuracy from, for example, 10^−6^ down to 10^−8^, costing mainly one division. It would be preferred over the optimization mentioned in “Symbolic iteration method based on computer algebra analysis (SICAA)” section. The starter of $E$ is shown in Eq. (3). We modify Eq. (4) into the symbolic form of the standard Newton–Raphson method, as shown in Eq. (12). The function $E_{n + 1} \left( {e,M} \right)$ in Eq. (12) is expanded at $e{ } = { }0$ by Taylor Series expansion in every operational step, shown in Eq. (13). The main schemes of the Newton-like SICAA method are shown in Eqs. (12) and (13).

11 $$E_{n + 1} = E_{n} - \frac{{f\left( {E_{n} } \right)}}{{f^{\prime}\left( {E_{n} } \right)}},\quad n = 0,1,2, \ldots$$

12 $$E_{n + 1} \left( {e,M} \right) \approx E_{n} \left( {e,M} \right) - \frac{{E_{n} \left( {e,M} \right) - M - e\sin E_{n} \left( {e,M} \right)}}{{1 - e\cos E_{n} \left( {e,M} \right)}}$$

13 $$E_{n + 1} \left( {e,M} \right) \approx E_{n + 1} \left( {0,M} \right) + E_{n + 1}^{^{\prime}} \left( {0,M} \right)e + \frac{{E_{n + 1}^{\prime \prime } \left( {0,M} \right)}}{2!}e^{2} + \cdots + \frac{{E_{n + 1}^{k} \left( {0,M} \right)}}{k!}e^{k}$$

where $$n = 0,1,2, \ldots \;k = 0,1,2, \ldots ,18$$.

### The Halley-like SICAA method

The Halley’s numerical iterative method has a cubic convergence, as shown in Eq. (14), while it is more sensitive to the starters. The starter of *E* is shown in Eq. (3). We modify Eq. (4) into the symbolic form of the Halley’s method, as shown in Eq. (15). The function $E_{n + 1} \left( {e,M} \right)$ in Eq. (15) is expanded at $e{ } = { }0$ by Taylor Series expansion in every step, shown in Eq. (16). The main scheme of the Halley-like SICAA method is shown in Eqs. (15) and (16).

14 $$E_{n + 1} = E_{n} - \frac{f}{{f^{\prime}}}\left[ {1 + \frac{{ff^{\prime \prime } }}{{2f^{\prime 2} }}} \right]\quad k = 1,2, \ldots$$

15 $$E_{n + 1} \left( {e,M} \right) \approx E_{n} - \frac{{E_{n} - M - e\sin E_{n} }}{{1 - e\cos E_{n} }}\left[ {1 + \frac{{\left( {E_{n} - M - e\sin E_{n} } \right)\left( {e\sin E_{n} } \right)}}{{2\left( {1 - e\cos E_{n} } \right)^{2} }}} \right]$$

16 $$E_{n + 1} \left( {e,M} \right) \approx E_{n + 1} \left( {0,M} \right) + E_{n + 1}^{\prime } \left( {0,M} \right)e + \frac{{E_{n + 1}^{^{\prime\prime}} \left( {0,M} \right)}}{2!}e^{2} + \cdots + \frac{{E_{n + 1}^{k} \left( {0,M} \right)}}{k!}e^{k}$$

where $$n = 0,1,2, \ldots \;k = 0,1,2, \ldots ,18$$.

**Figure 2:**
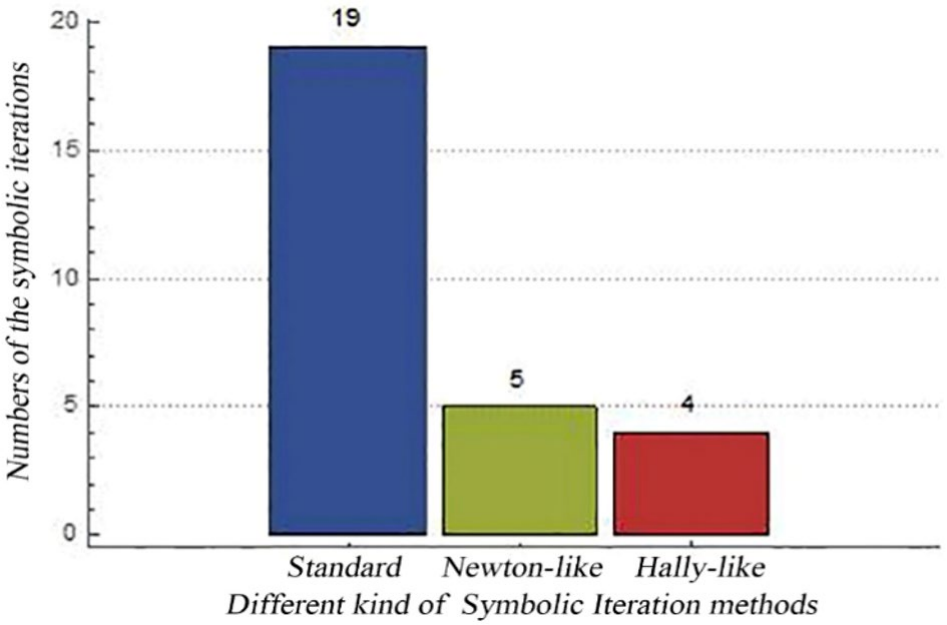
Number of iterations through standard SICAA method, Newton-like SICAA method and Halley-like SICAA method (double precision), until the *i*th iterative coefficients of $e^{n}$ $\left( {n = 1,2, \ldots ,18} \right)$ and the $\left( {i - 1} \right)$th iterative coefficients of $e^{n}$ $\left( {n = 1,2, \ldots ,18} \right)$ are the same.

When the coefficients of $\sin iM$ are the same between two symbolic iterations, which means $E_{n + 1} = E_{n}$, then $E_{n + 1}$ is regarded as the final analytic formula calculated by our SICAA methods. Figure 2 shows the number of symbolic iterations through three SICAA methods (double precision). As we can see, the standard SICAA method needs 19 steps to get the precision of $e^{18}$ series expansion, while the Newton-like SICAA method and the Halley-like SICAA method only need 5 steps and 4 steps, respectively. Although the final coefficients of $\sin iM$ calculated by any of these SICAA methods are the same, the Newton-like SICAA method has the advantages of both fewer symbolic operational steps than the standard SICAA method and simpler in symbolic form than the Halley-like one. Thus, we use the Newton-like SICAA method to get the final analytic formula.

## Analysis and comparison

### Accuracy and performance analysis

#### Analytic accuracy

**Figure 3:**
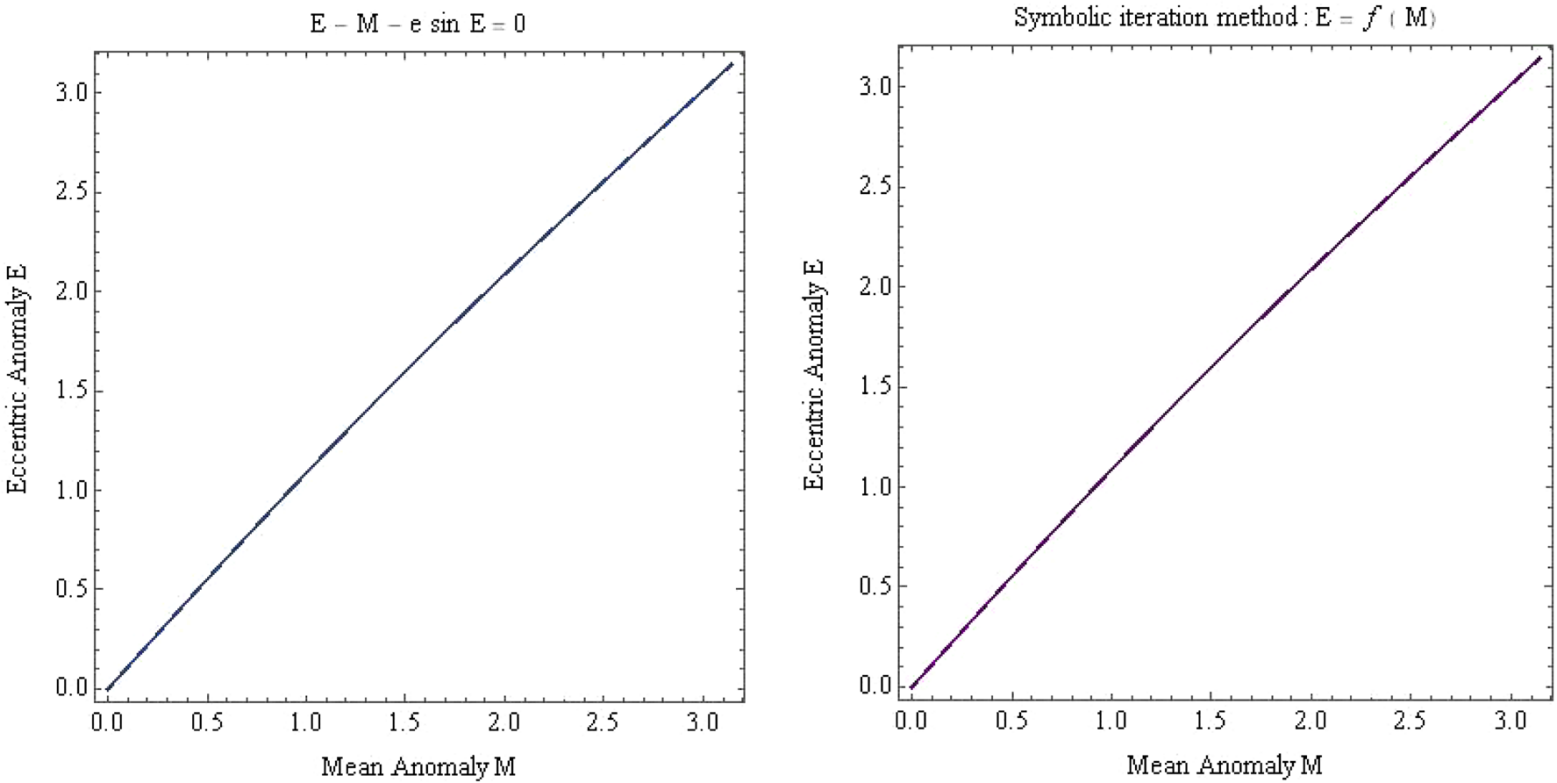
Explicit expression $E = f_{2} \left( M \right)$ of the SICAA method applied to the function $f_{1} \left( {M,E} \right) = E - M - 0.1\sin E$ over the domain $M \in \left[ {0, \pi } \right]$ (top right), matching nearly-circular orbit of eccentricity 0.1. The implicit function contour (top left) of the Kepler’s equation is shown for comparison.

After symbolic operation process, we change the implicit expression $f_{1} \left( {M,E} \right) = 0$ into the explicit expression $E = f_{2} \left( M \right)$, as shown in Eq. (6), thus obtaining the analytic solution of the Kepler’s equation when $e \ll 1$ to directly study the relationship between $E$ and $M$, as shown in Fig. 3.

**Figure 4:**
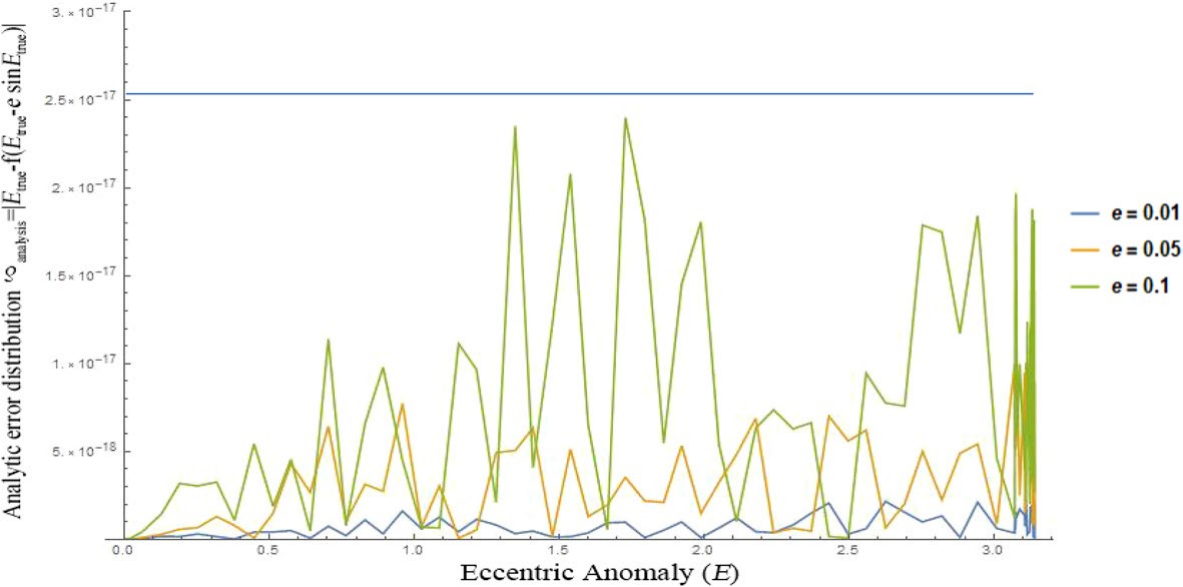
Analytic formula of the error distributions given by $\delta_{analysis} = \left| {E_{true} - f\left( {E_{true} - e\sin E_{true} } \right)} \right|$ over the domain $E \in \left[ {0,\pi } \right]$, matching nearly-circular orbits of eccentricity 0.01, 0.05, 0.1.

It is convenient to study the characteristics of the solution by explicit expression Eq. (6). To analyze the error properties, the error expression $\delta_{analysis}$ is defined, as shown in Eq. (17). The parameter *M* in Eq. (17) is calculated by the Eq. (18). After injecting Eqs. (6) and (18) into Eq. (17), the specific form of error $\delta_{analysis}$ is shown in Eq. (19), and the coefficient of $s_{i}$ is the same with that of the Eq. (6). Figure 4 shows the error distribution lines of the Eq. (19) over $E_{true} \in \left[ {0,\pi } \right]$ for the cases of eccentricity $e = 0.01, 0.05, 0.1$. As we can see in Fig. 4, the theoretical prediction of error $\delta_{analysis}$ for Eq. (6) is no more than 2.5 × 10^−17^ (shown by the blue line). Obviously, owing to the computer machine precision limits and truncation errors caused in every step, the numerical errors distribution will be slightly different with what are shown in Fig. 4, which will be discussed in the following part.

17 $$\delta_{analysis} = \left| {E_{true} - f_{2} \left( {e, M} \right)} \right|$$

18 $$M = E_{true} - e\sin E_{true}$$

19 $$\begin{aligned} \delta_{analysis} & = - e\sin E_{true} + a_{1} s_{1} + a_{2} s_{2} + a_{3} s_{3} + a_{4} s_{4} + a_{5} s_{5} + a_{6} s_{6} + a_{7} s_{7} + a_{8} s_{8} + a_{9} s_{9} + a_{10} s_{10} \\ & \quad + \;a_{11} s_{11} + a_{12} s_{12} + a_{13} s_{13} + a_{14} s_{14} + a_{15} s_{15} + a_{16} s_{16} + a_{17} s_{17} \\ \end{aligned}$$

where $$s_{i} = \sin \left( {iE_{true} - ie\sin E_{true} } \right)$$.

#### Numerical accuracy

**Figure 5:**
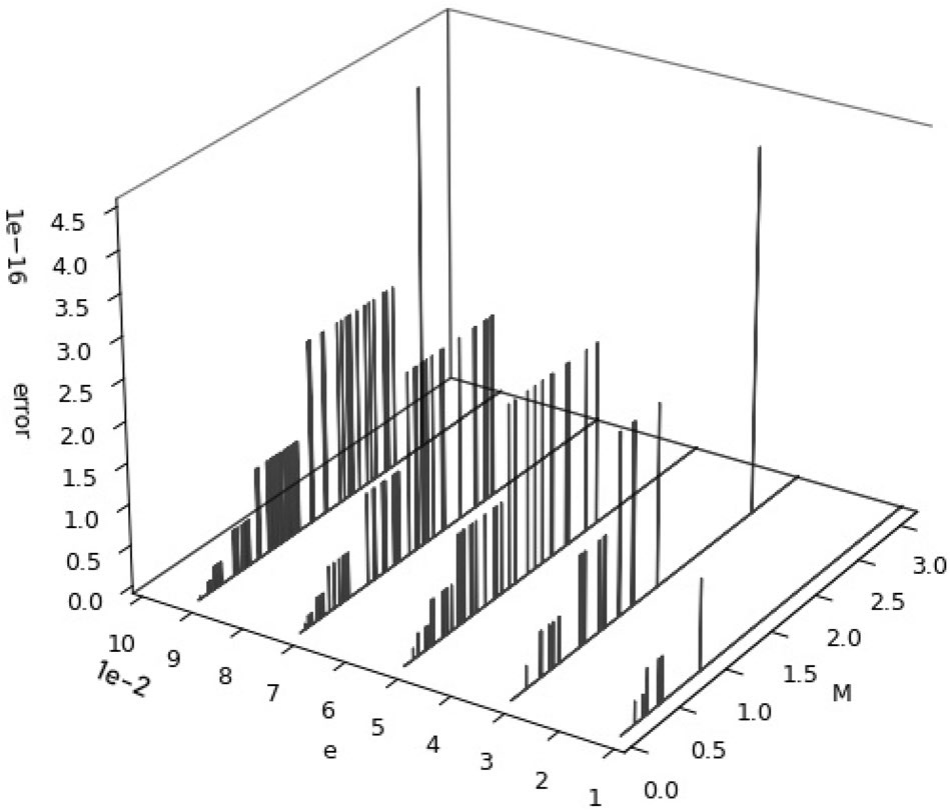
Numerical result of the SICAA method over $M \in \left[ {0, \pi } \right]$, matching nearly-circular orbits of eccentricity 0.01, 0.03, 0.05, 0.07, 0.09. The SICAA method is shown for 1001 × 5 grid points within the accuracy for $O\left( e \right)^{18}$, together with the evaluation of the numerical error: $\delta_{E} = \left| {E_{symbol} \left( M \right) - E} \right|$, where the error is computed by the method mentioned above.

To prove if the SICAA method can achieve double precision practically, we pre-calculated 1001 × 1001pairs of $\left( {E - e\sin E,{ }E} \right)$ uniformly sampled over $\left( {e,{ }E} \right) \in \left[ {0,0.1\left] \times \right[0,\pi } \right]$. The eccentric anomaly *E* is regarded as true values. Then, the $M\left( E \right) = E - e\sin E$ and *e* are the input of our algorithm, outputting the inverse function $E_{symbol} \left( M \right)$ as the solution of the Kepler’s equation. Differences between the true eccentric anomalies and the re-calculated ones $\delta_{E} = \left| {E_{symbol} \left( M \right) - E} \right|$ are shown in Fig. 5 for each case $e = 0.01, 0.03, 0.05, 0.07,0.09$. To better present the changing trends of the error $\delta_{E}$ with the increase of *M* and *e*, a waterfall picture of the error $\delta_{E}$ has been drawn in Fig. 5. With the increase of *M*, the errors increase and then decrease gradually, except for two outliers. With the increase of *e*, the errors increase slightly. Hence, the change of *e* has less influences on the precision in the SICAA method, while the accuracy of the SICAA solution is more sensitive to $M$.

**Figure 6:**
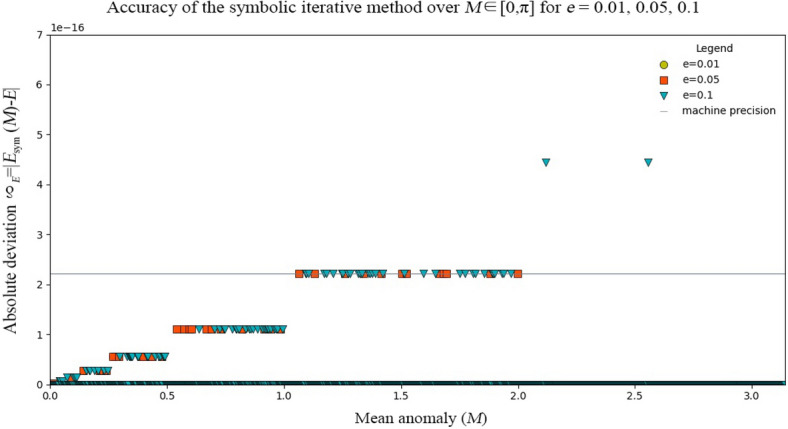
Accuracy of the SICAA method $\delta_{E} = \left| {E_{symbol} \left( M \right) - E} \right|$ as mentioned before over $M \in \left[ {0, \pi } \right]$ for the cases $e = 0.01, 0.05, 0.1$ with machine precision $\epsilon = 2.220446049250313 \times 10^{ - 16}$ by the grey line.

For $M \in \left[ {0, \pi } \right]$ and $e \in \left[ {0, 0.1} \right]$, most of the deviations $\delta_{E}$ are no more than machine precision $\epsilon = 2.220446049250313 \times 10^{ - 16}$ as indicated by the grey line in Fig. 6. As shown in Fig. 6, when $e = 0.1$, there are only two points higher than the machine accuracy $\epsilon$; when $e = 0.01$ and 0.05, all the errors are no more than the machine accuracy $\epsilon$; there are many errors even close to 0.

**Figure 7:**
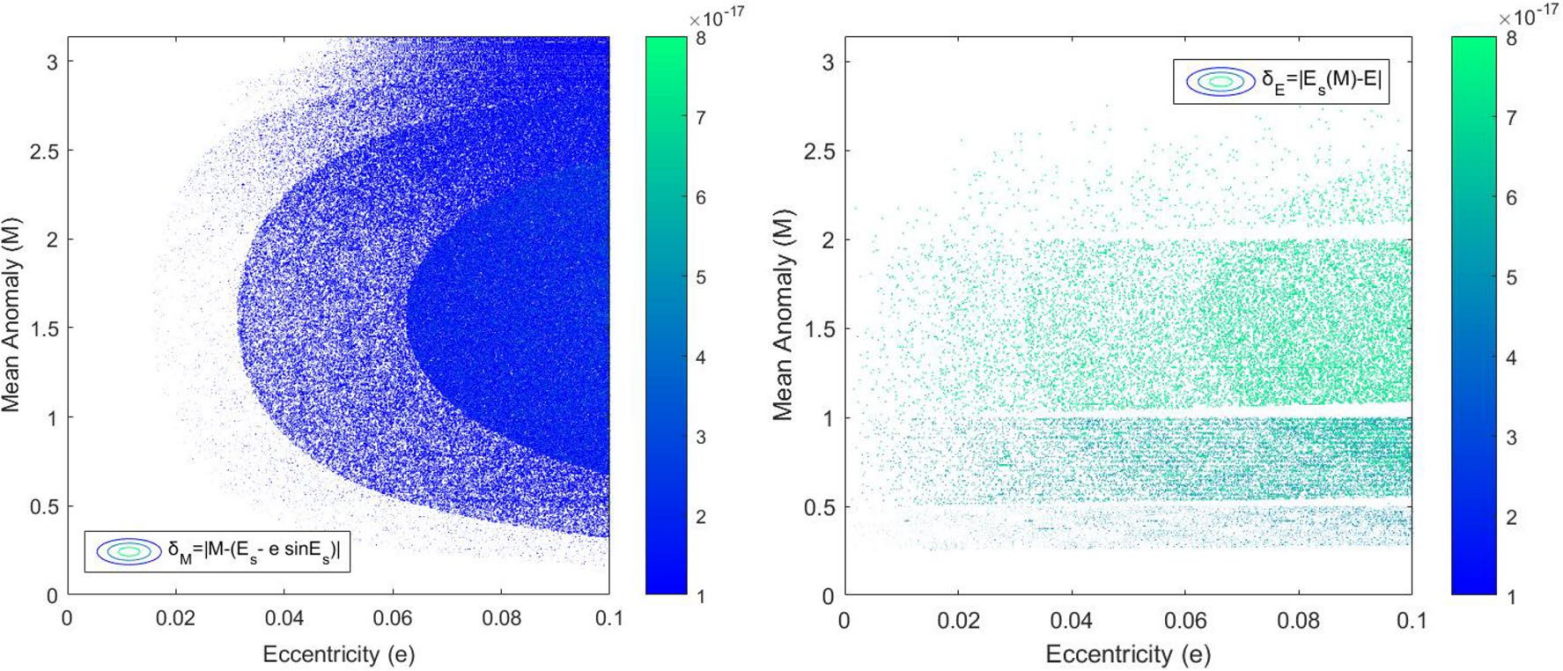
Contour plots of the errors $\delta_{M} = \left| {M - \left( {E_{symbol} - e\sin \left( {E_{symbol} } \right)} \right)} \right|$ (left plot) and $\delta_{E} = \left| {E_{symbol} \left( M \right) - E} \right|$ (right plot) over the domain $\left( {M,e} \right) \in \left[ {0,\pi } \right] \times \left[ {0,0.1} \right]$.

After $E_{symbol} \left( M \right)$ has been solved, we recomputed $M_{re}$ by $M_{re} = E_{symbol} - e\sin \left( {E_{symbol} } \right)$. Then, the contour plot of errors $\delta_{M} = \left| {M - M_{re} } \right| = \left| {M - \left( {E_{symbol} - e\sin \left( {E_{symbol} } \right)} \right)} \right|$ (left) (are shown in Fig. 7 over $\left( {M,e} \right) \in \left[ {0, \pi } \right] \times \left[ {0, 0.1} \right]$. It can be found from Fig. 7 (left) that as $M$ becomes closer to $\frac{\pi }{2}$, the contour lines become more and more dense, which means the error $\delta_{M}$ increases; with the increase of $e$, the color of contour lines changes from dark blue to light blue, which means the error $\delta_{M}$ increases slightly. That is matched perfectly with Fig. 5. As can be seen from Fig. 7 (right), the error value $\delta_{E}$ is symmetrically distributed with $M \approx \frac{\pi }{2}$; the more inclined it is to $\frac{\pi }{2}$, the greater the error $\delta_{E}$ is. With the increase of $e$, the error also increases slightly. However, the errors are almost all under $10^{ - 17}$.

Numerical errors (shown in Figs. 5, 6 and 7) almost match the analytic error distribution (shown in Fig. 4), with 99.93% of all numerical errors computed lower than machine precision $\epsilon$, and all $\delta_{E} = \left| {E_{symbol} \left( M \right) - E} \right| \le 4.4409 \times 10^{ - 16}$ over the entire interval.

#### Discussion of truncation error

**Figure 8:**
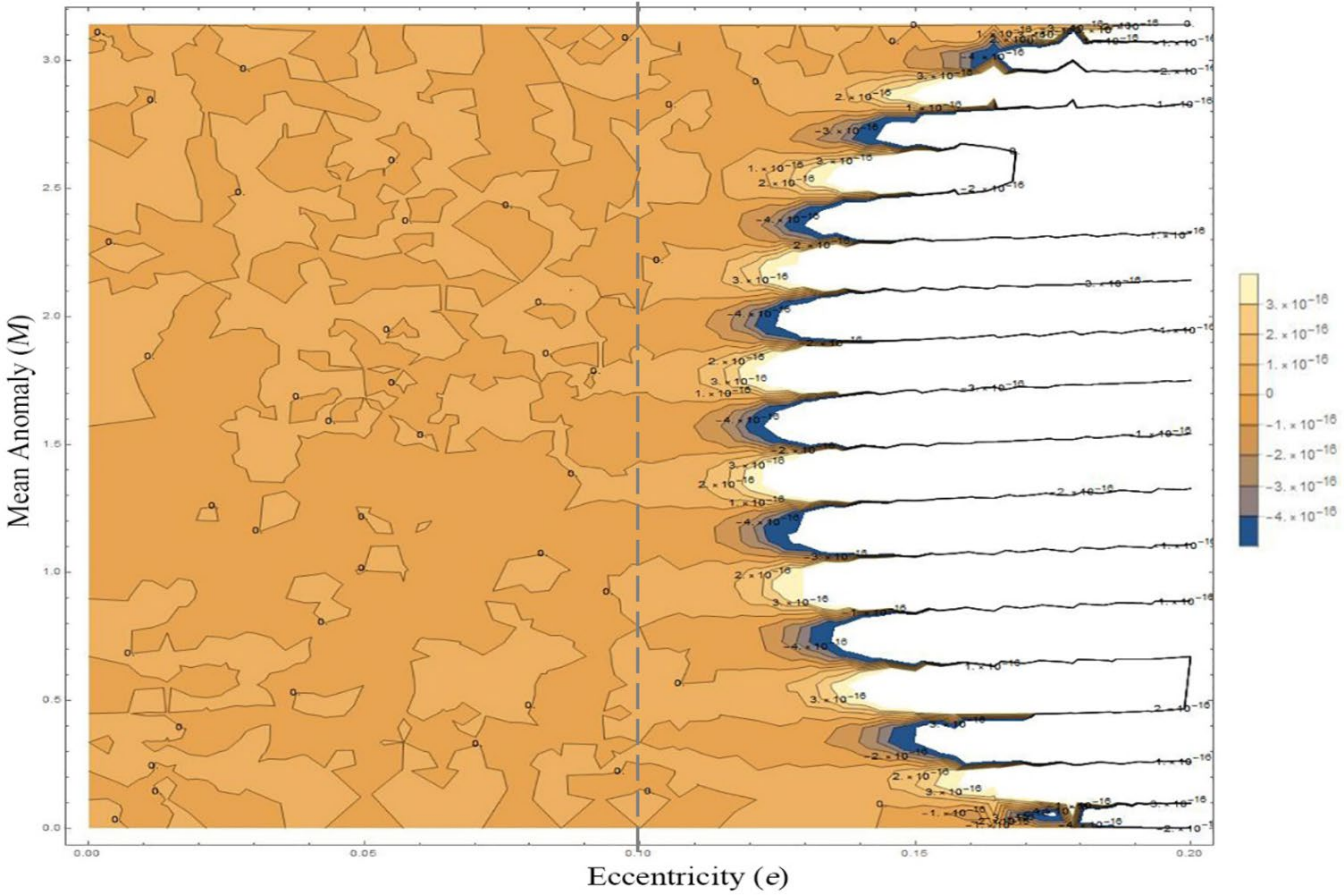
Contour plot of the errors $\delta_{analysis} = \left| {E_{true} - f\left( {E_{true} - e\sin E_{true} } \right)} \right|$ on the plane $\left( {e,E} \right) \in \left[ {0,0.2} \right] \times \left[ {0,\pi } \right]$. For visibility, the gray line shows the case $e = 0.1$.

The deviations $\delta_{E}$ and $\delta_{M}$ become larger in the corner $\left( {e \to 0.1,{ }M \to \frac{\pi }{2}} \right)$ of the plane $\left( {M,e} \right)$. That is because the Eq. (6) is expanded by Taylor series expansion at $e = 0$, also called Maclaurin series expansion. The principle of Maclaurin series expansion is that the equation is only available in the neighborhood of the expansion point $e = 0$, which is why when $e$ goes far away from 0, the errors become larger. Figure 8 shows the contour plot of the analytic errors $\delta_{analysis} = \left| {E_{true} - f_{2} \left( {E_{true} - e\sin E_{true} } \right)} \right|$ (as shown in Eq. (17)) on the plane $\left( {e,E} \right) \in \left[ {0,{ }0.2} \right] \times \left[ {0,{ }\pi } \right]$. In Fig. 8, the error $\delta_{analysis}$ increases significantly when $e = 0.2$ (strong elliptic), but it performs perfectly when $e \in \left[ {0,0.1} \right]$, meeting most needs of geoscience applications (most Earth satellites’ orbits are nearly-circular at $e = 10^{ - 2} \sim10^{ - 3}$).

To analyze the error distribution, we rearrange Eq. (6) as Eq. (20). The coefficients of $e^{i} { }\left( {i = 1,{ }2,{ } \ldots ,{ }18} \right)$ are expressed as $b_{i} \left( M \right)$. Table 1 represents the extremum value ${\text{Max}}\left( {b_{i} } \right)$ and the corresponding extremum point ${\text{Max}}\left( M \right)$ of the coefficient $b_{i} \left( M \right)$ over the domain $M \in \left[ {0,\pi } \right]$. Apparently, the coefficients of the $e^{n}$ are expressed in the polynomial function forms of $\sin iM$. Consequently, the larger the $b_{i}$ is, the larger the coefficients of $e$ are, and the greater the errors of $e$ accumulate, resulting in the larger final deviations. Therefore, when ${ }M \to \frac{\pi }{2}{ }$ and $e \to 1$, the error accumulated. The analytic error distribution $\delta_{analysis}$ is shown in Fig. 8. In spite of that, the errors shown in Fig. 8 are almost under $5 \times 10^{ - 16}$.

20 $$\begin{aligned} E\left( {e,M} \right) & = M + b_{1} e + b_{2} e^{2} + b_{3} e^{3} + b_{4} e^{4} + b_{5} e^{5} + b_{6} e^{6} + b_{7} e^{7} + b_{8} e^{8} + b_{9} e^{9} + b_{10} e^{10} \\ & \quad + b_{11} e^{11} + b_{12} e^{12} + b_{13} e^{13} + b_{14} e^{14} + b_{15} e^{15} + b_{16} e^{16} + b_{17} e^{17} + b_{18} e^{18} \\ \end{aligned}$$

**Table 1:** The Extremum values and the corresponding extremum point of the coefficients.

*b*_*i*_	*b*_1_	*b*_2_	*B*_3_	*b*_4_	*b*_5_	*b*_6_	*b*_7_	*b*_8_	*b*_9_	*b*_10_	*b*_11_	*b*_12_	*b*_13_	*b*_14_	*b*_15_	*b*_16_	*b*_17_
${\text{Max}}\left( {b_{i} } \right)$	1	0.5	0.5	0.46	0.5	0.59	0.7	0.89	1.18	1.47	2.0	2.56	3.54	4.66	6.51	7.25	12.30
${\text{Max}}\left( M \right)$	$\frac{\pi }{2}$	0.8	$\frac{\pi }{2}$	1.14	$\frac{\pi }{2}$	1.27	$\frac{\pi }{2}$	1.34	$\frac{\pi }{2}$	1.75	$\frac{\pi }{2}$	1.42	$\frac{\pi }{2}$	1.44	$\frac{\pi }{2}$	1.23	$\frac{\pi }{2}$

**Figure 9:**
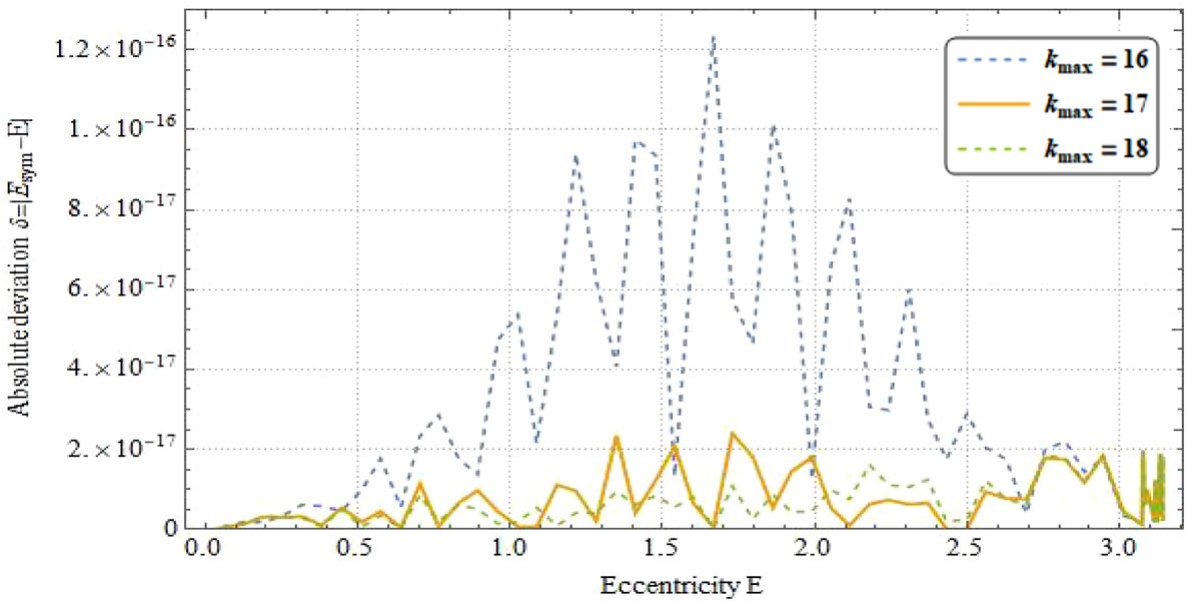
Errors $\delta_{analysis} = \left| {E_{true} - f\left( {E_{true} - e\sin E_{true} } \right)} \right|$ for the cases if the series is terminated at $k_{max} = 16$ (dashed-blue), $k_{max} = 17$ (solid), and $k_{max} = 18$ (dashed-green), respectively.

The plot of truncation driving errors are manifested in Fig. 9. The dashed-blue line pictures the errors $\delta_{analysis}$ when the series ceases at $k_{max} = 16$, which is lower than the machine precision $\epsilon$. With an additional term ($k_{max} = 17$), the errors $\delta_{analysis}$ are cut down to almost one order of magnitude lower than the ones at $k_{max} = 16$, portrayed by solid line. Then setting the termination at 18 ($k_{max} = 18$), represented by the dashed-green line, the errors $\delta_{analysis}$ are almost identical to the ones at $k_{max} = 17$, much lower than the standard double float accuracy, where we can get the tolerance of 2 × 10^−17^. Therefore, we use the Eq. (6) at $k_{max} = 17$ to run the numerical computation in the next section.

### Comparisons with traditional numerical iterative methods

In this section, error comparisons are conducted between our SICAA method and two traditional Newton iterative methods to deal with the Kepler’s equation. All methods were executed in the Python programming language, counting on numerical and algebraic libraries in the system. The details of the comparisons among our SICAA method, the standard Newton–Raphson method and the Halley’s method are as follows.

Hardware: Comparisons conducted on a modest laptop computer (Intel i7-8550U x64 CPU @ 1.8GHz 2GHz, RAM 8GB, and with the Windows operating system with 10.0.18362.1139 kernel).
Data preparation: Forward-calculating $M_{i,j} = E_{i} - e_{j} \sin E_{i}$, using 1001 × 1001 $\left( {E_{i} ,e_{j} } \right)$ pairs uniformly sampled by the equation $E_{i} = \frac{i}{1000}\pi$ and $e_{j} = 0.1 \times \frac{j}{1000}$, ($i,{ }j = 0,1, \ldots ,1000$). Then, the $M_{i,j}$ and $e_{j}$ are the input of our algorithm, outputting the inverse function $\widehat{{E_{i,j} }}\left( {M_{i,j} ,e_{j} } \right)$.
Precision: Average eccentric anomaly errors $\overline{\delta }_{E} \left( {e_{j} } \right){ }$ are computed by the Eq. (21), where $\delta_{Ei,j} \left( {M_{i,j} ,e_{j} } \right)$ denotes the error obtained in one computation, as shown in Eq. (22). The average CPU time (units: second) is computed by the Eq. (23), where $t_{i,j} \left( {M_{i,j} ,e_{j} } \right)$ denotes the CPU time cost in one computation. Starters: $E_{0} = M_{i,j}$, as mentioned in “Symbolic iteration method based on computer algebra analysis (SICAA)” section.
  21 $$\overline{\delta }_{E} \left( {e_{j} } \right) = \frac{{\mathop \sum \nolimits_{i = 0}^{1000} \delta_{Ei,j} \left( {M_{i,j} ,e_{j} } \right)}}{1001}$$
  22 $$\delta_{Ei,j} \left( {M_{i,j} ,e_{j} } \right) = \left| {f_{2} \left( {M_{i,j} ,e_{j} } \right) - \widehat{{E_{i,j} }}} \right|$$
  23 $$\overline{t}_{E} \left( {e_{j} } \right) = \frac{{\mathop \sum \nolimits_{i = 0}^{1000} t_{i,j} \left( {M_{i,j} ,e_{j} } \right)}}{1001}$$

**Figure 10:**
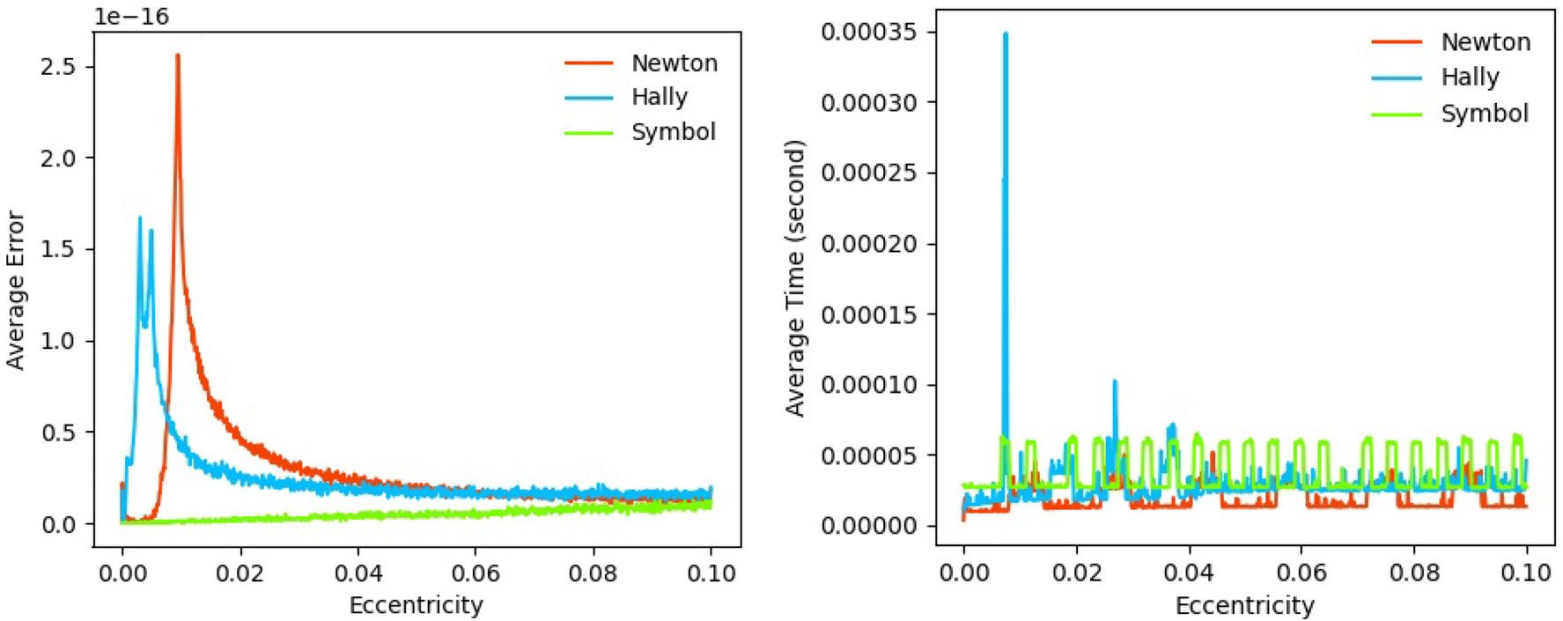
Numerical comparisons among our SICAA method, the standard Newton–Raphson method and the Halley’s method to solve the Kepler’s equation. Errors (left) and CPU times (right, units: second) are averaged with respect to $M \in \left[ {0, \pi } \right]$.

Figure 10 shows numerical comparisons among our SICAA method, the standard Newton–Raphson method and the Halley’s method. The SICAA method (Green line) has a higher precision than the traditional iterative methods when $e \ll 1$, with all errors under 4.5 × 10 − ^16^, less than machine precision $\epsilon$. As for CPU time cost, the SICAA method’ s time cost is close to the Halley’s method (cubic-Newton method, the blue line) at 10^−5^order of magnitude (units: second). Therefore, compared with traditional iterative methods, the SICAA method is available to solve the Kepler’ equation for nearly-circular orbits, by one order of magnitude more in precision with time cost at around 0.00005 s. The precision of our method is stable.

**Figure 11:**
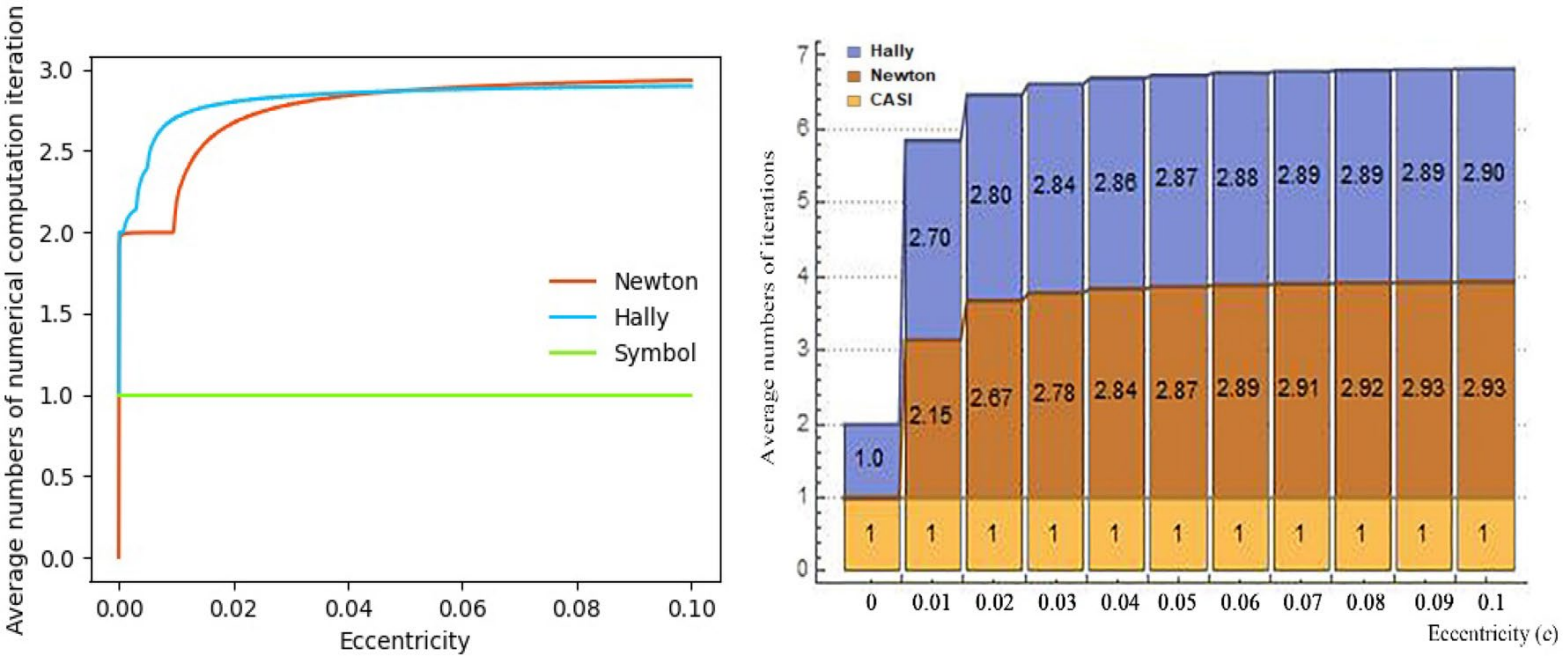
Compared with SICAA analytic solution, average numbers of iteration of standard Newton–Raphson method and Halley’s method, which are averaged with respect to $M \in \left[ {0, \pi } \right]$, respectively.

Compared with our analytic solution, Fig. 11 shows average numbers of iteration of two traditional Newton iterative methods. Our method only needs to solve one analytic formula to get the final results. With the increase of $e$, the iteration numbers of traditional methods increase rapidly, and the total computation numbers of transcendental functions will increase exponentially as iteration numbers grow. Meanwhile, iteration numbers are greatly affected by the iterative starters. When the quality of starters is poor, the performance of traditional iterative methods will be significantly degraded. Many studies^18^ focus on the starting algorithms of the traditional iterative methods to solve the Kepler’s equation. As for nearly-circular orbits, the starter $E_{0} = M$ is a simple form of starter with relatively small computational cost. However, compared with our method, there is still a certain gap in accuracy, mainly due to the limits of truncation caused by machine precision $\epsilon$ in each iterative step.

As can be seen from Fig. 6, 99.93% of the all errors computed by our SICAA method are lower than machine precision $\epsilon = 2.220446049250313 \times 10^{ - 16}$. The maximum errors are 4.4409 × 10^−16^ accounting for 0.07%, while minimum errors are 0 accounting for 96.42%, over the entire interval, showing that the accuracy is almost one order of magnitude higher than that of traditional Newton iterative methods (double precision). In this case, our method, to some extent, has a better accuracy with acceptable time consumption to solve the Kepler’s equation for nearly-circular motions than the traditional Newton iterative methods.

### Comparisons with traditional non-iterative methods

There are also many classic non-iterative solutions. For example, the method proposed by American scholar Nijenhuis^1^ in 1991 combines the Mikkola's idea and higher-order Newton's method. Besides, the method proposed by NASA researcher Markley^12^ in 1995 is based on higher-order processing of cubic algebraic equations. At that time, those methods could effectively solve the Kepler’s equation, by reducing the calculation numbers of the transitional functions and providing non-iterative solutions, which optimizes the algorithms, compared with the Newton iterative methods. However, due to the limitations of computer science at that time, these methods could not balance the accuracy with CPU time consumption. With the rapid development of computer science, although their ideas still shine with wisdom, the algorithms themselves are difficult to meet the current requirements of accuracy.

## Discussions and applications

### Mathematics essence of our method and relationship with traditional Fourier–bessel series

The traditional series expansion of the eccentric anomaly is provided by scientists Colwell^13^ and Battin^9^, as shown in Eqs. (24) and (25).

24 $$E = M + 2\mathop \sum \limits_{k = 1}^{\infty } \frac{1}{k}J_{k} \left( {ke} \right)\sin kM$$

25 $$J_{k} \left( {ke} \right) = \mathop \sum \limits_{j = 0}^{\infty } \left( { - 1} \right)^{j} \frac{{\left( \frac{ke}{2} \right)^{k + 2j} }}{{j!\left( {k + j} \right)!}}$$

The Eq. (24) contains two infinite series, which cannot be directly solved by numerical methods. The scientists have studied the truncation methods of infinite Fourier–Bessel representations, for example, the method based on the Lambert W function^17^ (referred to as the Lambert W method), as shown in Eqs. (26) and (27). Moreover, the kth-order Bessel function also need to be truncated, as shown in Eq. (28), where $j_{max} = s$ is obtained by the Eqs. (29) and (30).

26 $$E \approx M + 2\mathop \sum \limits_{k = 1}^{{k_{max} }} \frac{1}{k}J_{k}^{s} \left( {ke} \right)\sin kM$$

27 $$k_{max} = k^{*} - 1 = \left( {\frac{{c_{2} }}{{c_{1} }}} \right){\mathcal{W}}\left( {{\text{exp}}\left( {\frac{{c_{3} }}{{c_{2} }}} \right)\frac{{c_{1} }}{{c_{2} }}} \right) - 1$$

28 $$J_{k}^{s} \left( {ke} \right) = \mathop \sum \limits_{j = 0}^{s} \left( { - 1} \right)^{j} \frac{{\left( \frac{ke}{2} \right)^{k + 2j} }}{{j!\left( {k + j} \right)!}}$$

29 $$s = \left( {\frac{{k_{max} }}{2}} \right) + 2\frac{{g_{1} \left( {\frac{{k_{max} }}{2}} \right)}}{{g_{2} \left( {\frac{{k_{max} }}{2}} \right)}}$$

30 $$\left\{ {\begin{array}{*{20}l} {g_{1} \left( {s^{*} } \right) = \frac{d}{{ds^{*} }}\left( {\frac{1}{{g\left( {s^{*} } \right)}}} \right)} \hfill \\ {g_{2} \left( {s^{*} } \right) = \frac{{d^{2} }}{{ds^{*2} }}\left( {\frac{1}{{g\left( {s^{*} } \right)}}} \right)} \hfill \\ \end{array} } \right.$$

In Eq. (27), $c_{1}$, $c_{2}$, $c_{3}$ are parameters related to the basic inequality of kth-order Bessel function^24^ and the Lambert W function^17^.

**Figure 12:**
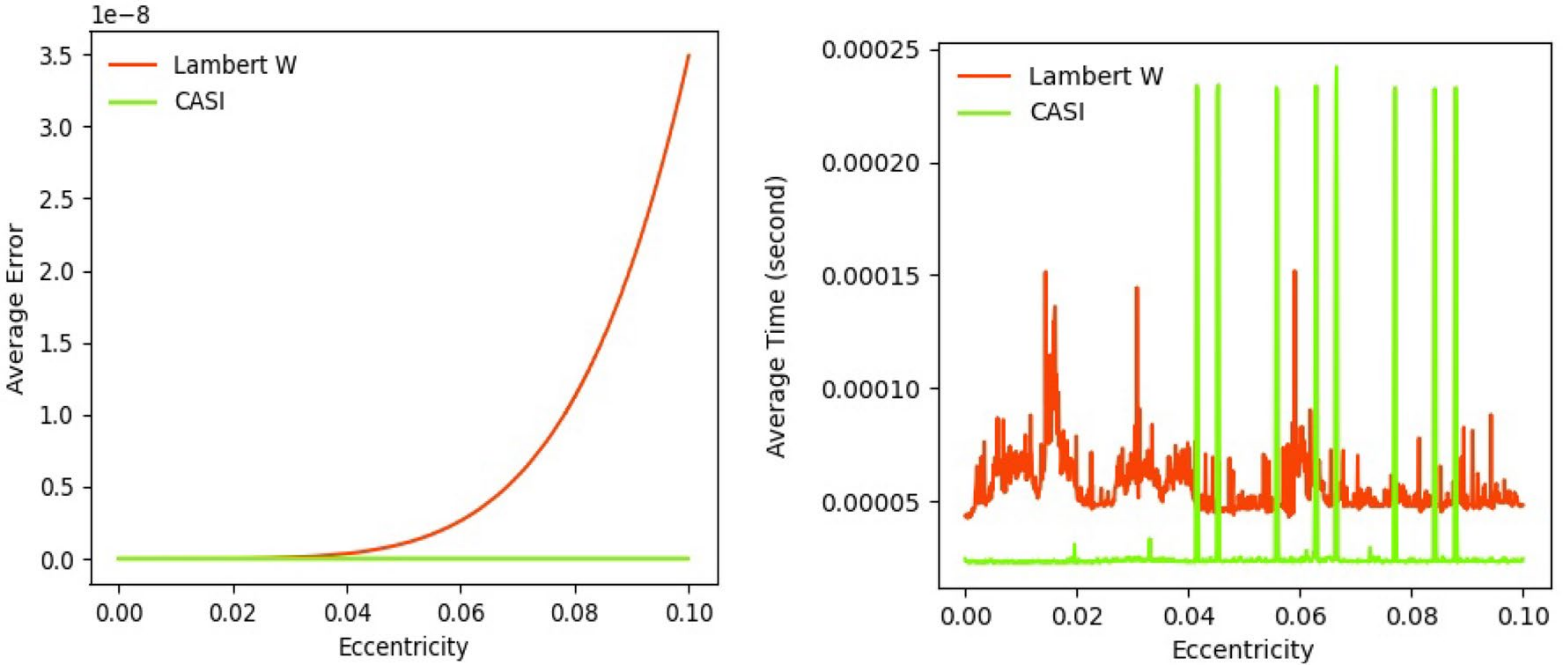
Numerical comparisons among the SICAA method and the Lambert W method to solve the Kepler’s equation. Errors (left) and CPU times (right, units: second) are averaged with respect to $M \in \left[ {0, \pi } \right]$ over the domain $e \in \left[ {0, 0.1} \right]$.

As our method can reach the accuracy of 10^−17^ (given in “Analysis and comparison” section), we compare the truncation of infinite series expansion based on the Lambert W function (the Lambert W method) at the same accuracy of 10^−17^. For $e = 0.1$ and $10^{{ - N_{tol} }} = 10^{ - 17}$, the max order obtained through the Eq. (27) ^17^ is $k_{max} = 17$. To reach the accuracy of 10^−17^ for a second-order Newton–Raphson solution, we use $k = k_{max} = 17$, and $e = 0.1$ in the Eq. (28) and the Eq. (29); the order needed in $J_{17} \left( {17e} \right)$ is $s = 1$. Figure 12 shows numerical comparisons between the SICAA method and the Lambert W method. The SICAA method (Green line) has a higher precision than the Lambert W method. As for CPU time cost, the SICAA method’s time cost is shorter than that of the Lambert W method except for a few outliers. Therefore, compared with the traditional truncation of series expansion method, our method presents better truncation combinations of the two infinite series expansion for solving the Kepler’s equation when the orbit is nearly-circular.

In conclusion, based on the Eq. (9) and the Eq. (10), the mathematics essence of our method is a new effective and accurate truncation method of Fourier–Bessel representations for the Kepler’s equation, which could achieve the accuracy of $E$ at $10^{ - 17}$, with higher accuracy than traditional truncation method of Lambert W’s.

### The performance and the application of the SICAA method

At present, most of the solutions for the Kepler’s equation focus on refining the iterative methods. Relying on high-speed computer hardware, by optimizing the iterative algorithms and their starters, iterative methods^25^ are often more efficient to solve transcendental functions than complex analytic solutions, not to mention that there are still many transcendental functions that could not be solved analytically. For example, in 2019, the fast switch and spline scheme^21^ proposed by Spanish scholar Daniele Tomasini is used to solve the Kepler’s equation with a faster speed than that of the standard Newton–Raphson method. The CORDIC-like iterative method^20^ proposed by German scholar Zechmeister can be applied for both elliptic orbits and hyperbolic orbits, and consumes less CPU time than the Newton iterative methods. However, If only nearly-circle orbits are considered, our method could get accuracy at $10^{ - 17}$, the accuracy of which is higher than CORDIC-like iterative method.

## Conclusion

This paper presents a new efficient and accurate truncation method of the infinite Fourier–Bessel representation, symbolic iteration method based on computer algebra analysis, taking Kepler’s equation as an example. Based on the computer algebra system, it eventually provides an analytical formula to compute the eccentric anomaly without complex iterative computation at run-time. Compared with traditional truncation method (Lambert W), our method has higher accuracy and shorter CPU consumption; compared with commonly used iterative methods (the standard Newton–Raphson method and the Halley’s method), our method has higher accuracy and stronger robustness; compared with high-performance iterative algorithms proposed in the new century, on the basis of retaining the accuracy of the algorithm, our method takes into account the advantages of analytical methods and the simplicity of codes. Further, the simple codes make our method well-suited for various satellite orbit determination algorithms, semi-analytic satellite orbit propagators form low fidelity to high fidelity, and other geoscience fields.

## Supplementary Information

Supplementary Information.

## Data Availability

Name of code: “CASI Mthod.py” and “numerical CASI.py”; Developer: Ruichen Zhang; Year first available: 2020.11; Hardware required: Win 10; Software required: Mathematica 11.2; Python 3.8; Program language: Mathematica, python; Program size: 4 KB, 1 KB; The source code are available for download at GitHub: https://github.com/chur-614/CSI_chur.
