# Supplementary material for: Symbolic iteration method based on computer algebra analysis for Kepler’s equation
Source: Sci Rep. 2022 Feb 22;12:2957. doi: 10.1038/s41598-022-07050-5 (PMC8863892; doi:10.1038/s41598-022-07050-5)
Supplement: Supplementary file 1 — Supplementary Information. [file 41598_2022_7050_MOESM1_ESM.pdf]

S.1 Algorithm steps of SICAA method

Algorithm 1 Algorithm steps of SICAA method

Step 1: Starter	In[1]:= $E_0 = M$
1st step	In[2]:= $E_1 = M + e\,Sin[E_0]$
2nd step	In[3]:= $E_2 = FullSimplify[Series[M + e\,Sin[E_1], \{e, 0, \alpha\}]]$
3rd step	In[3]:= $E_3 = FullSimplify[Series[M + e\,Sin[E_2], \{e, 0, \alpha\}]]$
…	… *(until coefficients of* $\sin iM$ *are the same between two iterations)*
End	$E_{19} = E_{18}$

S.2 Algorithm steps of Newton-like SICAA method

Algorithm 2 Algorithm Steps of Newton-like SICAA method

Step 1: Starter	In[1]:= $E_0 = M$
1st step	In[2]:= $E_1 = FullSimplify[Series[E_0 - \frac{E_0 - M - eSin[E_0]}{1 - eCos[E_0]}, \{e, 0, \alpha\}]]$
2nd step	In[3]:= $E_2 = FullSimplify[Series[E_1 - \frac{E_1 - M - eSin[E_1]}{1 - eCos[E_1]}, \{e, 0, \alpha\}]]$
…	… *(until coefficients of* $\sin iM$ *are the same between two iterations)*
End	$E_5 = E_4$

S.3 Algorithm steps of the Hally-like SICAA

Algorithm 3 Algorithm steps of the Hally-like SICAA

Step 1: Starter	In[1]:= $E_0 = M$
1st step	In[2]:= $E_1 = Simplify[Series[E_0 - \frac{E_0 - M - e\sin E_0}{1 - e\cos E_0}\left[1 + \frac{(E_0 - M - e\sin E_0)(e\sin E_0)}{2(1 - e\cos E_0)^2}\right], \{e, 0, \alpha\}]]$
2nd step	In[3]:= $E_2 = Simplify[Series[E_1 - \frac{E_1 - M - e\sin E_1}{1 - e\cos E_1}\left[1 + \frac{(E_1 - M - e\sin E_1)(e\sin E_1)}{2(1 - e\cos E_1)^2}\right], \{e, 0, \alpha\}]]$<br>Out[3]:= …
…	… *(until coefficients of* $\sin iM$ *are the same between two iterations)*
End	$E_4 = E_3$

S. 4 Coefficients of $\sin nM$ based on SICAA method

a_k	**Coefficients of $\sin nM$ $(n = 1, 2, \ldots, k)$**
a_i	$\varepsilon\, e^{i} - \beta\, e^{i+2} + \gamma\, e^{i+4} - \cdots + \delta\, e^{i+2s} + O[e]^{i+2s}$ $(s = 0,1,2 \ldots)$
